# Oculogyric Crisis after Initiation of Aripiprazole: A Case Report of an Active Duty Service Member

**DOI:** 10.1155/2023/9440028

**Published:** 2023-01-10

**Authors:** Nicole L. Hadler, Yevin A. Roh, David A. Nissan

**Affiliations:** Department of Mental Health Services, Naval Medical Center San Diego, San Diego, CA 92134, USA

## Abstract

**Introduction:**

Oculogyric crisis is an acute dystonic reaction characterized by sustained, bilateral, and upward deviation of the eyes. It is a relatively uncommon extrapyramidal side effect of antipsychotic medications. Aripiprazole is an atypical antipsychotic that is FDA-approved for the treatment of schizophrenia, bipolar disorder, Tourette's disorder, and treatment resistant major depressive disorder. Compared to other antipsychotics, it is thought to have a lower propensity for causing dystonic side effects. *Clinical Case*. This case report is of a 19-year-old male who was psychiatrically hospitalized for first episode psychosis and initiated on low-dose oral aripiprazole. Three days after initiation of the medication, the patient was found to be markedly anxious and pacing around his room. Exam was notable for intermittent upward eye rolling, sustained upward conjugate gaze, and limited downward gaze. No other facial dyskinetic movements were observed. *Treatment*. The patient's oral aripiprazole was held, and he was administered 50 mg of oral diphenhydramine with improvement in symptoms within one hour. Ocular symptoms, dizziness, frontal headache, and pacing were resolved the following morning. He declined reinitiation of an antipsychotic medication.

**Conclusion:**

Aripiprazole-induced acute dystonia, specifically OGC, is a rare potential adverse effect of aripiprazole. Risk factors include male gender, young age, use of typical antipsychotics, and initiation or uptitration of an antipsychotic. Even though atypical antipsychotics including aripiprazole are associated with lower risk for extrapyramidal symptoms, the possibility of oculogyric dystonia merits close monitoring especially in young, male, and drug naive patients. Anticholinergic agents such as diphenhydramine can treat symptoms of acute dystonia.

## 1. Introduction

Aripiprazole is an atypical antipsychotic medication used in the treatment of schizophrenia, bipolar disorder, and treatment resistant depression. It is associated with several potential adverse effects, including metabolic side effects such as weight gain and dyslipidemia as well as Parkinsonism, tardive dyskinesia, akathisia, neuroleptic malignant syndrome, and acute dystonia such as oculogyric crisis [1, 2]. Treatment of adverse effects associated with aripiprazole involves adjusting the antipsychotic dosage [3] as well as medication intervention to target the specific adverse effect: a beta blocker for akathisia [2], a VMAT2 inhibitor such as tetrabenazine for tardive dyskinesia [4], and benztropine or diphenhydramine for acute dystonia [5, 6]. Oculogyric crisis (OGC) is an acute dystonic reaction characterized by sustained, bilateral, and upward deviation of the eyes [3, 6]. Episodes can last seconds to hours [6, 7], and it is often preceded by anxiety or emotional lability [8]. Associated symptoms can include restlessness, agitation, malaise, fixed stare, pain, increased blinking, and neck dystonia [9]. OGC induced by aripiprazole, a dopamine partial agonist, is particularly uncommon [10].

## 2. Case Description

A 19-year-old previously healthy male was psychiatrically hospitalized for first episode psychosis. After an unremarkable medical workup, the treatment team initiated 5 mg of oral aripiprazole for treatment of a presumed primary psychotic disorder. Three days later, he was found to be markedly anxious and pacing around his room on the psychiatric unit. When interviewed, he reported bilateral eye pain, frontal headache, and anxiety. On exam, he was anxious appearing and had difficulty sitting still in the chair. He was closing his eyes and rubbing his temple and forehead region with his hands. Exam was also significant for intermittent upward conjugate eye rolling, sustained upward conjugate gaze, and limited downward gaze. The treating team noted no other facial dyskinetic movements.

The treatment team held the patient's oral aripiprazole, and he consented to being administered 50 mg of oral diphenhydramine with improvement in symptoms within one hour. Ocular symptoms, frontal headache, and pacing were completely resolved the following morning. Given that his pacing resolved concomitantly with the resolution of oculogyric symptoms, the team opted that psychotropic treatment for akathisia was not indicated. He declined reinitiation of an antipsychotic medication. He declined additional dosages of diphenhydramine.

## 3. Discussion

Aripiprazole-induced acute dystonia, specifically oculogyric crisis, is a rare potential adverse effect of aripiprazole and atypical antipsychotics in general [7, 9, 11]. Diagnosis of OGC is clinical. Slow and Lang propose a list of required criteria: tonic, conjugate ocular deviation; duration of minutes to hours; preservation of consciousness [8]. Supportive features include that the episode is preceded by anxiety and/or discomfort, the patient is aware of and bothered by the ocular symptoms, association with other dystonia, association with a low dopaminergic state, and improvement with an anticholinergic or dopaminergic medication [8]. Other associated symptoms can include worsening of hallucinations or delusions, paranoia, obsessive thoughts, agitation, and catatonia [12]. It is critical to distinguish these symptoms as due to OGC versus due to the patient's primary psychiatric disorder given the opposing treatment approaches—withholding antipsychotics in the former and augmenting antipsychotic treatment in the latter [6, 12, 13]. The most common etiology of OGC is drug induced, including antipsychotics, antiemetics, antiepileptics, selective serotonin reuptake inhibitors, and tricyclic antidepressants[9, 14]. A number of neurologic and neurodegenerative disorders have also been implicated in OGC, including acute herpetic brainstem encephalitis, neurosyphilis, Wilson's disease, and Rett's syndrome [9]. Risk factors for OGC include male gender, younger age, use of first generation antipsychotic, initiation or uptitration of an antipsychotic, and personal or family history of acute dystonia [7, 9, 14]. The pathophysiology of OCG is thought to occur due to dysfunction in dopaminergic neurotransmission and an acute hypodopaminergic state induced by antipsychotic medications, though an imbalance of dopaminergic and cholinergic transmission may also play a role [9, 14]. In this case, the patient developed OCG shortly after initiation of an antidopaminergic medication, which quickly resolved with administration of diphenhydramine, consistent with the hypothesis of dopamine deficiency and relative cholinergic overactivity [8]. OCG is treated by stopping or discontinuing the offending agent and administering an anticholinergic such as benztropine or an antihistamine with anticholinergic properties, such as diphenhydramine [9, 14]. Benztropine and diphenhydramine also have the benefit of having intramuscular formulations if the severity of the patient's distress or dystonia prevents them from accepting oral medications [13]. It is recommended that medication treatment be continued for one week to prevent recurrence. When restarting an antipsychotic medication, providers should consider an antipsychotic with a lower risk of dystonic side effects, such as quetiapine or clozapine [7].

This case highlights an uncommon side effect that can be caused by any antipsychotic medication, regardless of the potency of dopaminergic blockade. While not fatal, oculogyric crisis can cause significant distress and discomfort and place the patient at increased risk of treatment nonadherence [6, 7]. Therefore, any patient being initiated on an antipsychotic should be closely monitored for acute dystonic reactions, to include OGC, especially if they carry additional risk factors such as younger age and male gender. Furthermore, OGC should be treated promptly by adjusting the culprit medication and/or administering an antihistaminergic or anticholinergic medication.
